# Supplementary material for: Exapted CRISPR-Cas12f homologs drive RNA-guided transcription
Source: Nature. Author manuscript; Available in PMC 2026 Aug 13. (PMC13472631; doi:10.1038/s41586-026-10166-7)
Supplement: Supplementary file 1 [file NIHMS2195280-supplement-dCas12f_Supp_Fig1v3.pdf]

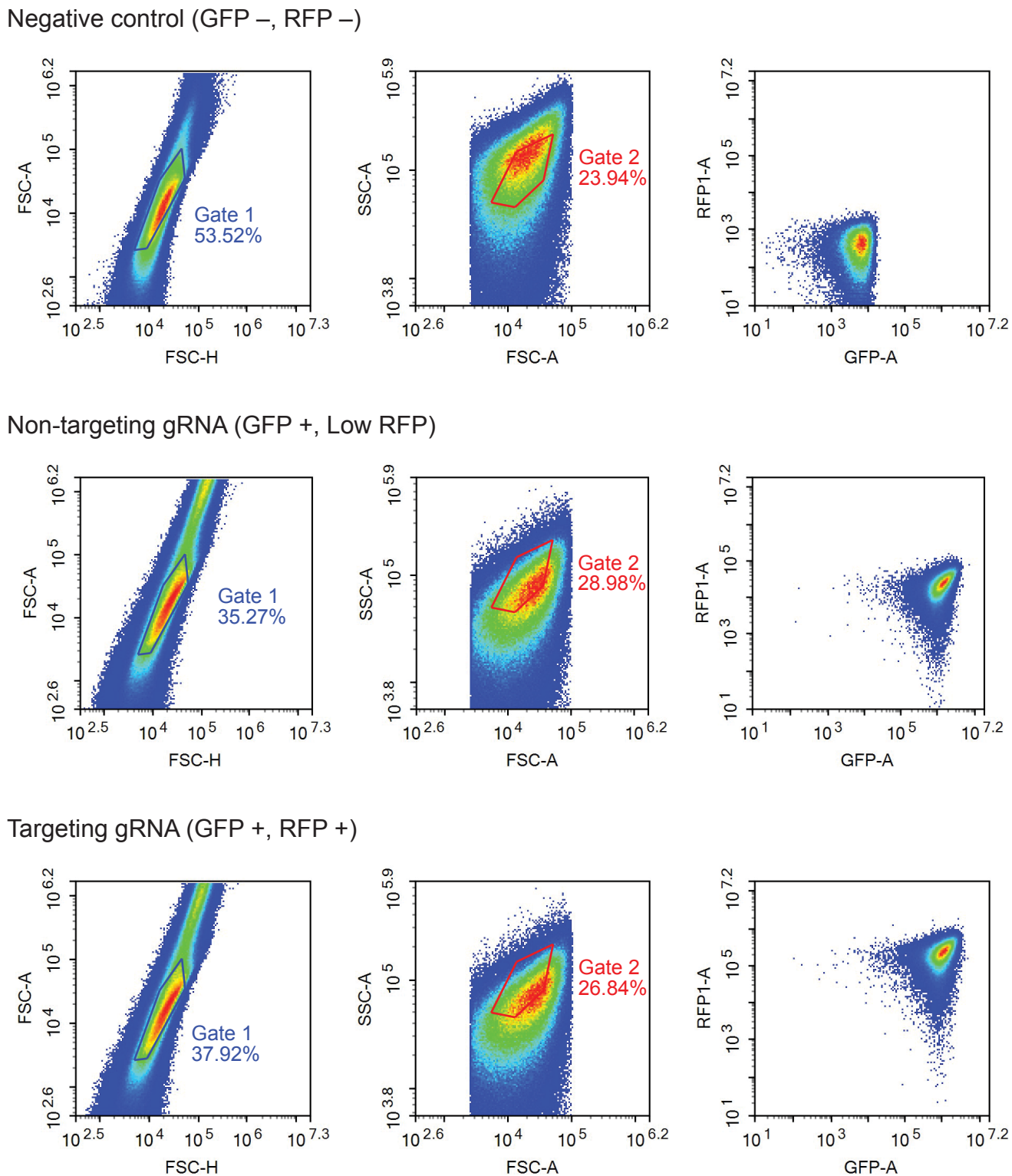
Negative control (GFP –, RFP –)
FSC-A
FSC-H
Gate 1
53.52%
SSC-A
FSC-A
Gate 2
23.94%
RFP1-A
GFP-A
Non-targeting gRNA (GFP +, Low RFP)
FSC-A
FSC-H
Gate 1
35.27%
SSC-A
FSC-A
Gate 2
28.98%
RFP1-A
GFP-A
Targeting gRNA (GFP +, RFP +)
FSC-A
FSC-H
Gate 1
37.92%
SSC-A
FSC-A
Gate 2
26.84%
RFP1-A
GFP-A
