# Supplementary material for: Exapted CRISPR-Cas12f homologs drive RNA-guided transcription
Source: Nature. Author manuscript; Available in PMC 2026 Aug 13. (PMC13472631; doi:10.1038/s41586-026-10166-7)
Supplement: Supplementary file 3 [file NIHMS2195280-supplement-Hoffmann_etal_SIGuide.docx]

**Supplementary Information**

**Exapted CRISPR-Cas12f homologs drive RNA-guided transcription**

Florian T. Hoffmann^1^, Tanner Wiegand^1,2^, Adriana I. Palmieri^1^, Juniper Glass-Klaiber^3^, Renjian Xiao^4^, Stephen Tang^1^, Hoang Le^1^, Chance Meers^1,2*^, George D. Lampe^1,2^, Leifu Chang^4,5^, Samuel H. Sternberg^1,2†^

^1^Department of Biochemistry and Molecular Biophysics, Columbia University, New York, NY, USA.

^2^Howard Hughes Medical Institute, Columbia University, New York, NY, USA.

^3^Department of Cellular Physiology and Biophysics, Columbia University, New York, NY, USA.

^4^Department of Biological Sciences, Purdue University, West Lafayette, IN, USA.

^5^Purdue Institute for Cancer Research, Purdue University, West Lafayette, IN, USA.

^*^Current address: Department of Biochemistry, Vanderbilt University, Nashville, TN, USA.

^†^Corresponding author. Email: shsternberg@gmail.com

**Supplementary Figures**

Supplementary Figure 1 | Representative gating schemes for flow cytometry analysis of RFP fluorescence reporter.

**Supplementary Tables**

Supplementary Table 1 | Cas12f homologs in Fig. 1b phylogenetic tree.

Supplementary Table 2 | dCas12f, HTH, and σ^E^ homologs in Fig. 1e phylogenetic trees.

Supplementary Table 3 | σ^E^ homologs in Fig. 1f phylogenetic tree.

Supplementary Table 4 | List of dCas12f-σ^E^ systems, proteins, and gRNAs tested in this study.

Supplementary Table 5 | Strains used in this study.

Supplementary Table 6 | Description and sequence of plasmids used in this study.

Supplementary Table 7 | Oligonucleotides used in this study.

Supplementary Table 8 | Sequencing files and information for this study.
